# Mild antiresorptive activity of an anti-vascular endothelial growth factor A antibody and sunitinib in a rat model of bone resorption

**DOI:** 10.1016/j.bonr.2025.101837

**Published:** 2025-03-17

**Authors:** J.I. Aguirre, S.M. Croft, E.J. Castillo, C.J. Cruz-Camacho, D.B. Kimmel

**Affiliations:** Department of Physiological Sciences, University of Florida (UF), Gainesville, FL, United States of America

**Keywords:** Medication-related osteonecrosis of the jaw, Antiresorptives, Aangiogenesis inhibitors, Schenk assay, Bone histomorphometry, Sunitinib, Anti-VEGFA antibody

## Abstract

Medication-Related-Osteonecrosis-of-the-Jaw (MRONJ) is an adverse event linked to antiresorptives such as bisphosphonates and denosumab. While MRONJ predominantly affects cancer patients treated with these agents, it has been less frequently reported in cancer patients receiving angiogenesis inhibitors (AgIs) like bevacizumab and sunitinib, even without concurrent use of antiresorptives. We *hypothesized* that certain AgIs exhibit antiresorptive activity in addition to their antiangiogenic effects, potentially influencing the pathophysiology of MRONJ.

52 five-week-old SD rats were randomized to receive vehicle (VEH), an oncologic dose of zoledronic acid (ZOL), or low (LD) and high doses (HD) of either an anti-VEGFA antibody or sunitinib (SU) for 10 days. We used the Schenk assay to assess the *in vivo* antiresorptive properties of these drugs/agents. We evaluated serum biomarkers of bone resorption (TRACP 5b) and formation (P1NP), pQCT variables of the femurs/tibias, and bone resorption/formation variables by bone histomorphometry at the distal femur metaphysis.

ZOL reduced TRACP-5b levels, osteoclast number, and BFR while increasing vBMD, mineralized tissue volume, calcified cartilage volume, and bone volume. Both anti-VEGFA and SU decreased osteoclast number and increased calcified cartilage volume relative to total mineralized tissue volume, though to a lesser extent than ZOL. Anti-VEGFA (HD) also reduced TRACP-5b levels. Furthermore, both AgIs decreased P1NP levels, MAR, and bone elongation rate but increased growth cartilage thickness and induced physeal dysplasia.

In conclusion, AgIs, particularly anti-VEGFA, exhibit significant yet milder antiresorptive activity compared to ZOL. They also affect bone formation, suggesting a complex mechanism that may play a role in the pathophysiology of MRONJ.

## Highlights

• An oncologic dose of ZOL is a strong positive control to validate the Schenk assay.
• Anti-VEGFA and SU, particularly the former, exhibit mild antiresorptive activity.
• Anti-VEGFA and SU decrease osteoclast number and increase calcified cartilage volume.
• Anti-VEGFA and SU reduce serum P1NP levels and decrease MAR and bone elongation.
• Anti-VEGFA and SU (HD) induce growth plate thickening and physeal dysplasia.

## 1 Introduction

Medication-related osteonecrosis of the jaw (MRONJ) is a potentially serious adverse event characterized by exposed bone or bone that can be probed through intraoral or extraoral fistula(e) in the maxillofacial region, persisting for at least eight weeks in patients receiving powerful antiresorptives (pARs), either alone or in combination with immune modulators or angiogenesis inhibitors (Ruggiero et al., 2022). pAR drugs associated with MRONJ include the nitrogen-containing bisphosphonates (N-BPs) zoledronic acid (ZOL), alendronate, pamidronate, ibandronate, minodronate, and risedronate (Khosla et al., 2007; Bilezikian, 2006; Marx, 2003; Khan et al., 2015), and the anti-RANKL antibody denosumab (Stopeck et al., 2010; Van den Wyngaert et al., 2011). In patients with specific types of cancer, high cumulative doses of pARs are indicated as adjuvant therapy for controlling hypercalcemia of malignancy and skeletal-related events (SREs), including bone metastases (Stopeck et al., 2010; Van den Wyngaert et al., 2011; Van Poznak et al., 2017; Aapro et al., 2008; Ibrahim et al., 2003). Conversely, in patients with osteoporosis, low cumulative doses of pARs are given as first-line treatment to decrease the risk of fragility fracture and improve bone density (Black et al., 2000; Black et al., 2012; Adler et al., 2016). Though MRONJ is common in pAR-treated patients with certain types of cancer (1.8–5 %), it is rare in pAR-treated patients with osteoporosis (0.01–0.03 %) (Ruggiero et al., 2022; Khosla et al., 2007; Khan et al., 2015; Rugani et al., 2016; Coleman et al., 2018; Van Poznak et al., 2021). Furthermore, while MRONJ is most commonly associated with pARs, it has also been reported in pAR-naïve cancer patients receiving anticancer drug combinations, including systemic angiogenesis inhibitors (AgIs), such as the anti-vascular endothelial growth factor A (VEGFA) antibody *bevacizumab* (BVZ), and multitargeted receptor tyrosine kinase inhibitors (TKIs), such as sunitinib (SU) and dasatinib (Aragon-Ching et al., 2009; Nicolatou-Galitis et al., 2019; Pimolbutr et al., 2018; Koch et al., 2011; Katsenos et al., 2012; Disel et al., 2012; Estilo et al., 2008; Greuter et al., 2008; Guarneri et al., 2010; Serra et al., 2009). However, the incidence of MRONJ in pAR-naïve cancer patients has been reported as 0.3–0.4 %, tending to be lower than that in cancer patients treated only with pARs (Guarneri et al., 2010).

Several hypotheses have been advanced to explain the pathophysiology of MRONJ, including inhibition of bone resorption, presence of inflammation/infection, inhibition of angiogenesis, innate or acquired immune dysfunction, and genetic factors (Ruggiero et al., 2022; Ruggiero et al., 2014; Aghaloo et al., 2015). Of these, both the inhibition of bone resorption and the presence of inflammation/infection are considered the leading hypotheses (Ruggiero et al., 2022; Khosla et al., 2007; Bilezikian, 2006; Aguirre et al., 2021). These combined hypotheses suggest that tooth-related local risk factors in the oral cavity (*e.g.*, extraction of infected teeth, periodontal infection, and periapical infection) lead to associated inflammation and infection, resulting in the death of bone cells in the affected areas of the alveolar or jaw bone. When coupled with a systemic risk factor (*e.g.*, concurrent administration of pARs) that inhibits the removal of dead bone, the sequence of events creates a critical mass of necrotic bone that accumulates and eventually becomes exposed (Ruggiero et al., 2022; Aguirre et al., 2021). While this hypothesis explains the role of pARs, it does not explain the involvement of other drugs, such as AgIs, as systemic risk factors.

While AgIs are primarily recognized for their powerful inhibition of angiogenesis (Mendel et al., 2003; Willett et al., 2004; Ferrara, 2004), certain AgIs linked to MRONJ, such as anti-VEGFA, SU, and dasatinib, have also been shown to inhibit osteoclast formation and activity *in vitro* (Maita et al., 2012; Murray et al., 2003; Davis et al., 2011; Miyazaki et al., 2004; Park et al., 2019; Lowe et al., 1993). Additionally, they affect osteoclast differentiation and function, suggesting some degree of anti-resorptive activity *in vivo* (Maita et al., 2012; Niida et al., 2005; Niida et al., 1999). These effects are attributed to their targeting of various receptors, including VEGFRs, PDGFRs, CSF1R (Maita et al., 2012; Murray et al., 2003; Davis et al., 2011; Patyna et al., 2008), and c-SRC (Mendel et al., 2003; Miyazaki et al., 2004; Park et al., 2019; Le Tourneau et al., 2007; Zhao and Adjei, 2015; Qin et al., 2019; Xin et al., 2009; O'Farrell et al., 2003; Zhang et al., 2014). All these data together suggest that AgIs may play a role in MRONJ pathophysiology through not only their anti-angiogenic effects but also an understudied *in vivo* antiresorptive mechanism.

AgIs are rarely administered as monotherapy. They are typically combined with other anti-neoplastic agents, including chemotherapy drugs such as taxanes (docetaxel, paclitaxel), alkylating agents (cisplatin, oxaliplatin), antimetabolite pyrimidine antagonists (5-fluorouracil, capecitabine, and gemcitabine) (Katsenos et al., 2012; Disel et al., 2012; Estilo et al., 2008; Greuter et al., 2008; Guarneri et al., 2010; Serra et al., 2009), as well as pARs (Fusco et al., 2015; Beuselinck et al., 2012), and/or glucocorticoids (Nicolatou-Galitis et al., 2019; Pimolbutr et al., 2018; Estilo et al., 2008; Guarneri et al., 2010). Consequently, most clinical data regarding MRONJ associated with AgIs come from cancer patients receiving multiple medications, which complicates the ability to determine the specific role of AgIs in MRONJ pathogenesis.

Currently, limited preclinical studies have focussed on the potential role of AgIs in the pathophysiology of MRONJ (Messer et al., 2020; Akita et al., 2018; Zhao et al., 2023). Given the increasing number of AgIs in use and development, particularly the anti-VEGFA antibody BVZ and its biosimilars, along with TKIs such as SU, gaining a deeper understanding of the role of AgIs in MRONJ pathophysiology represents a critical unmet medical need.

To address this gap, we applied a traditional *in vivo* rat model to investigate the impact of two AgIs on bone resorption and related bone activities (Miller and Jee, 1975; Schenk et al., 1986; Schenk et al., 1973). We hypothesized that anticancer drugs like BVZ and certain TKIs exhibit unique *in vivo* anti-resorptive activity, which could influence bone resorption in the context of MRONJ.

## 2 Materials and methods

### 2.1 Animal care and management

We utilized 52 five-week-old male Sprague-Dawley (SD) rats (Charles River; Madison, WI, USA), weighing an average of 120 g on arrival. Rats were kept in pairs under standard laboratory conditions with a 13-h light/11-h dark cycle, a constant temperature of 25 °C, a humidity of 48 % and unlimited access to water and food (Envigo Harlan 2018 Rat Diet, Madison, WI, USA). All animal procedures were approved by the Institutional Animal Care and Use Committee at the University of Florida (Gainesville, FL, USA). The Animal Care Services at the University of Florida (UF) is an AAALAC-accredited animal care and use program.

### 2.2 Study design

**Supplemental Fig. 1:**
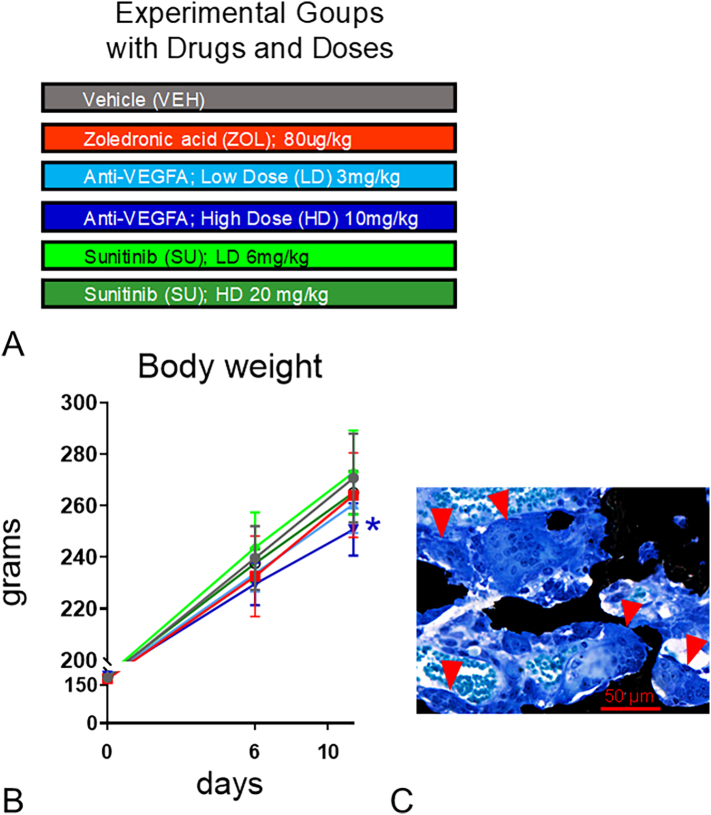
**Experimental design, body weight and giant osteoclasts:** (**A**) **Experimental Design**. A total of 52 male Sprague-Dawley (SD) rats were randomized into the following groups to start the study at age 6 weeks. A vehicle (***VEH***) group (*n* = 12 rats) received either saline (*n* = 4) intravenously (IV), an IgG solution IV (n = 4), or 10 % DMSO/90 % PEG 300 solution (n = 4) by oral gavage. A ***ZOL*** group (*n* = 8 rats) received 80 μg/kg/IV/bolus of zoledronic acid on day 1 of the study. An ***anti-VEGFA*** (***LD***) group (n = 8 rats) treated with a low dose (3 mg/kg) of anti-VEGFA (B20–4.1.1) monotherapy IV on days 1, 4, and 7, respectively; an ***anti-VEGFA*** (***HD***) group (n = 8 rats) treated with a high dose (10 mg/kg) of anti-VEGFA (B20–4.1.1) monotherapy IV on days 1, 4, and 7, respectively; a ***SU*** (***LD)*** group (n = 8 rats) treated with a low dose (6 mg/kg) of sunitinib dissolved in 10 % DMSO/90 % PEG 300 daily by oral gavage (n = 8 rats); and a ***SU*** (***HD)*** group (n = 8 rats) treated with a high dose (20 mg/kg) of sunitinib dissolved in 10 % DMSO/90 % PEG 300 daily by oral gavage, were also included; (**B**) **Body weight** was taken at baseline (day 0), day 6 and day 10 (necropsy day). *Significantly different from the VEH control group (*P* < 0.05); (**C**) **Giant osteoclasts** are depicted with red arrowheads. These giant osteoclasts have many nuclear profiles and were sometimes seen at the trabecular surfaces of ZOL treated rats but not in the rats of other groups. von Kossa/tetrachrome stain.

One week was given to the rats after arrival for acclimation. The rats were then weighed and randomized to conduct a 10-day study. Supplemental Fig. 1A depicts the experimental groups. Rats in the vehicle (***VEH***) group (*n* = 12) received either saline (*n* = 4) intravenously (IV), an IgG solution (n = 4) IV, or 10 % DMSO/90 % PEG 300 solution (n = 4) by oral gavage. Rats in the ***ZOL*** group (*n* = 8/group) received an 80 μg/kg/IV/bolus of zoledronic acid (ZOL; Novartis Pharma AG; Basel, Switzerland) on Day 1. ZOL was dissolved in sterile saline (pH 7.2, 0.2 mg/ml) and injected at 0.4 ml/100 g body weight into the tail vein of each rat. Rats in the ***anti-VEGFA*** low dose (***LD***) and high dose (***HD***) groups (n = 8/group) received either 3 or 10 mg/kg IV of anti-VEGFA (B20–4.1.1) on days 1, 4, and 7, respectively. On the injection day, B20–4.1.1 (17.26 mg/ml) was diluted to working stocks in saline (pH 7.2). *Bevacizumab* (BVZ) is an AgI that specifically targets and blocks human VEGFA. Therefore, we utilized the B20–4.1.1 antibody (Genentech, South San Francisco, CA, USA), a rodent surrogate of BVZ that blocks both human and rodent VEGFA. This antibody was designed for use in rat and murine studies producing VEGFA serum concentrations comparable to those observed in patients receiving BVZ (Messer et al., 2020; Bagri et al., 2010; Jiang et al., 2014; MacMillan et al., 2014; Boult et al., 2017; English et al., 2017; Lai et al., 2017; Sampath et al., 2013; Liang et al., 2006). A dose of 5 mg B20–4.1.1/kg mimics in rodents the pharmacokinetic profile of BVZ in patients with cancer (Bagri et al., 2010; Lu et al., 2008; Han et al., 2016). Rats in the ***SU*** groups received either 6 or 20 mg SU/kg (***LD*** and ***HD***) (*n* = 8/group) in 10 % DMSO/90 % PEG 300 daily by oral gavage (Bagi et al., 2009; Kappers et al., 2010). The SU dose/schedule is based on published studies (Patyna et al., 2008; Bagi et al., 2009; Singh et al., 2012; Speed et al., 2012; Blasi et al., 2012; Tugues et al., 2007). All rats were injected SC with declomycin or calcein (Sigma Chemical Co., St Louis, MO, USA) at a dose of 15 mg/kg on Days 5 and 1 before sacrifice, respectively, to label sites of bone formation (Aguirre et al., 2001; Aguirre et al., 2012; Aguirre et al., 2007).

Body weight measurements were taken on Days 0, 6, and 10. On the necropsy day (Day 10), rats were exposed to CO_2_ for >1 min to induce a rapid unconscious state and death. During the unconscious state, a cardiac puncture was performed with a 2 cc syringe on each rat to yield ~1–1.5 ml of whole blood. Then, death was confirmed by conducting a bilateral thoracotomy. Blood in the 2 cc syringe was allowed to clot at room temperature and then centrifuged at 3000 RPM for 10 min. The serum was then collected and stored at −20 °C. The obtained blood serum was used to determine circulating tartrate-resistant acid phosphatase 5b [(TRAPC5b); IDS, Scottsdale, AZ] (Yarrow et al., 2023) and procollagen type 1 N-terminal propeptide [(P1NP); Elabscience Biotechnology Inc., Houston, TX] (Cheng et al., 2021), as whole-body bone resorption and formation markers, respectively.

The right femurs and tibiae were dissected free, trimmed free of fat and muscle, and stored at −20 °C. Subsequently, peripheral quantitative computed tomography (pQCT) was performed to assess various pQCT variables at the distal femoral and proximal tibial metaphyses (details below). The left femur and tibiae were dissected, trimmed free of fat and muscle, and fixed in 10 % neutral buffered formalin for 48 h before being transferred to 70 % ethanol. The left femurs were then sectioned transversely at the midshaft using a Dremel Moto-Tool (Dremel; Racine, WI, USA), dehydrated in ethanol, and embedded undecalcified in methyl methacrylate (Baron et al., 1983). Frontal plane methyl-methacrylate femur sections of the distal metaphysis of the left femur were prepared with a microtome (Leica/Jung 2265, Leica Biosystems; Deer Park, IL, USA) to assess the anti-resorptive effects of each drug utilizing the Schenk assay (see below) and bone histomorphometry (see below), by evaluating skeletal variables related to bone resorption and formation.

***Peripheral Quantitative Computed Tomography (pQCT) analyses*** were conducted at the distal femoral and proximal tibial metaphyses, as previously described (Ke et al., 2001; Messer et al., 2018). Briefly, the right femurs and tibiae were thawed to room temperature. They remained wrapped in saline-soaked gauze except during scanning with a Stratec XCT Research M instrument (Norland Medical Systems; Fort Atkinson, WI, USA), using manufacturer's software version 5.40. At the distal femur, three transverse scans (voxel size = 0.10 mm × 0.10 mm × 010 mm) were taken at the secondary *spongiosa* at levels corresponding to 25, 30 and 35 % of the total bone length with a starting reference line at the border of the intercondylar notch of the distal epiphysis. Three transverse scans were taken at the secondary spongiosa of the proximal tibia at levels corresponding to 20, 25 and 30 % of the total bone length with a starting reference line at the border of the tibial plateau. In both the distal femur and proximal tibial metaphyses, the 20–25 % levels are closer to the growth plate, whereas the 30–35 % levels are closer to the diaphysis. Total metaphyseal bone variables, which include both cortical and trabecular bone, were total bone mineral content (total BMC, mg/mm), total volumetric bone mineral density (total vBMD, mg/cm^3^), and total bone area (mm^2^). Trabecular metaphyseal bone variables, including trabecular BMC (trabecular BMC, mg/mm) and volumetric trabecular (vBMD, mg/cm^3^), were determined in a central area bounded by the endocortical surface at the distal femoral and proximal tibial metaphyses. Data of all pQCT variables were reported as the mean of the three levels measured in each metaphysis of each rat.

### 2.3 Bone tissue processing

Trabecular bone histomorphometry, growth plate thickness and rate of bone elongation were analyzed at the distal femur using sections embedded in methyl-methacrylate. The blocks were sectioned in the frontal plane at 4- and 8-μm thicknesses with a microtome (Leica/Jung 2265, Leica Biosystems; Deer Park, IL, USA). A few 4 μm bone sections were stained with toluidine blue, as well as von Kossa counterstained with tetrachrome stain (Polysciences, Inc., Warrington, PA, USA) for the structural and osteoclast number variables. The 8 μm sections remained unstained to measure fluorochrome-based indices of bone formation for calculation of dynamic histomorphometric variables and the rate of bone elongation.

### 2.4 The Schenk assay model

This model was specifically designed to test the capacity of compounds/drugs to inhibit the resorption of mineralized tissues *in vivo* (Miller and Jee, 1975; Schenk et al., 1986; Schenk et al., 1973). It is performed in growing rats at trabecular bone regions within the metaphyses of long bones. It relies on normal physiological processes of the growth plate and the metaphysis associated with bone elongation, which includes both bone resorption and bone formation. Metaphyseal trabeculae in growing rats are composed of two types of mineralized tissue: calcified cartilage and woven bone. Furthermore, in rapidly growing rats, the metaphysis is a site of net loss of hard tissues resulting from the combined processes of resorption of calcified cartilage and woven bone by osteoclasts and the formation of woven bone by osteoblasts. When bone and/or calcified cartilage resorption is inhibited, as occurs with pARs, the total amount of calcified cartilage and trabecular bone in the metaphysis rises (Miller and Jee, 1975; Schenk et al., 1986; Schenk et al., 1973), giving a reasonable quantitative index of resorption activity.

We used frontal sections of the distal femoral metaphysis to analyze several variables within a metaphyseal region of interest (ROI). The ROI for the Schenk assay (ROI1) included both the primary and secondary *spongiosa* and was enclosed in an area from a line along the proximal border of the growth plate [growth cartilage-metaphyseal junction (GCMJ)] to a perpendicular line 1.5 mm proximal to the GCMJ and 0.25 mm inside each endocortical surface. For the evaluation, we utilized two methods: 1) undecalcified sections stained with von Kossa to determine the volume of mineralized tissue (mm^3^), which is analogous to the bone volume fraction (BV/TV) often reported in conventional bone histomorphometry, and 2) consecutive undecalcified sections stained with toluidine blue. This metachromatic stain selectively differentiates between calcified cartilage and woven bone tissue. Thus, we employed toluidine blue to quantify the volume of calcified cartilage tissue (mm^3^), the volume of trabecular bone tissue (mm^3^), and the percentage of calcified cartilage tissue volume relative to the total mineralized tissue volume (%).

### 2.5 Bone histomorphometry

Trabecular bone histomorphometry variables were assessed using standard histomorphometry techniques with the Osteomeasure System (Osteometrics, Decatur, GA, USA) within ROI2 at the distal femoral metaphysis (Aguirre et al., 2001; Iwaniec et al., 2007). All data were collected by a single investigator blinded to the identity of each section under analysis. ROI2 was defined as an area within the *secondary spongiosa*, extending from 0.4 mm (to exclude the primary *spongiosa*) to 1.5 mm from the growth plate and 0.25 mm inside the endocortical surface. Structural parameters were measured in von Kossa-stained sections at 40× magnification. They included trabecular bone surface (BS, mm), trabecular thickness (Tb.Th, μm), trabecular separation (Tb.Sp, μm) and trabecular number (Tb.N, #/mm). Osteoclast number (Oc.N/BS, #/mm), the number of osteoclasts per millimeter of the bone surface, was measured using von Kossa-stained sections at 200× magnification. Dynamic histomorphometry was evaluated using fluorochrome-based indices of trabecular bone formation in 8 μm thick, unstained sections at 200× magnification under fluorescent illumination by identifying single-labeled surface (sLS, mm) and double-labeled surface (dLS, mm). Mineralizing surface (MS/BS, %), an index of active bone formation, was calculated as the percentage of the total trabecular bone surface that displayed a double-labeled surface plus one-half the single-labeled surface. Mineral apposition rate (MAR; μm/day), an index of osteoblast activity, was calculated by dividing the interlabel distance at double-label sites by 4, the time interval (d) between the administration of fluorochrome labels. Surface-based bone formation rate (BFR/BS; μm^3^/μm^2^/day) was calculated by multiplying MS/BS by MAR (Frost and Recker, 1983). Our terminology was based on recommendations of the Histomorphometry Nomenclature Committee of the American Society of Bone and Mineral Research (Dempster et al., 2013; Parfitt et al., 1987).

### 2.6 Growth cartilage thickness and rate of bone elongation

The thickness of the growth cartilage at the distal femur, defined as the distance along the longitudinal axis from the distal end of the growth cartilage (facing the subchondral bone) to the GCMJ, was measured at six sites in each bone. The mean of the six sites was taken as the growth cartilage thickness (GC·Th, μm). Bone elongation rate was assessed in 8 μm unstained undecalcified distal femur metaphyseal sections at 100× magnification. The bone elongation rate was calculated by measuring the distance between the GCMJ and the most recent fluorescent calcein band (Day 1) parallel to the GCMJ at six equally spaced sites per section at 200× magnification. The mean distance was then divided by the time interval between the administration of calcein and the day of euthanasia (1 day) (Li et al., 1996).

### 2.7 Statistics

The data for all variables were analyzed to assess the significance of differences between the VEH group and the various agents, as well as among the anti-angiogenic agents in the ZOL group. For normally distributed data, we utilized one-way ANOVA with Dunnett's multiple-planned comparisons for *post hoc* analysis. In cases where the dataset did not follow a normal distribution, such as for the histomorphometric variables (LS/BS, dL/BS, MS/BS, MAR, BFR/BS, growth plate thickness, and bone elongation rate), we applied the non-parametric Kruskal-Wallis test followed by Dunn's multiple comparison test. A *p*-value of <0.05 was considered statistically significant. Data are expressed as mean ± SD unless otherwise indicated.

## 3 Results

### 3.1 General observations and body weight

All rats completed the study. Because there were no significant differences in any of the analyzed variables among the three control subsets [IV saline (*n* = 4), IV IgG solution (n = 4), and oral 10 % DMSO/90 % PEG 300 (n = 4)], their data were consolidated into a single VEH control group (*n* = 12). At Days 0 and 6, no differences in body weight were found among the groups. On Day 10, the anti-VEGFA (HD) group had significantly lower body weight than the VEH-treated group (*P* = 0.0042) (Supplemental Fig. 1B).

### 3.2 TRAcP 5b serum levels and osteoclast number

**Fig. 1:**
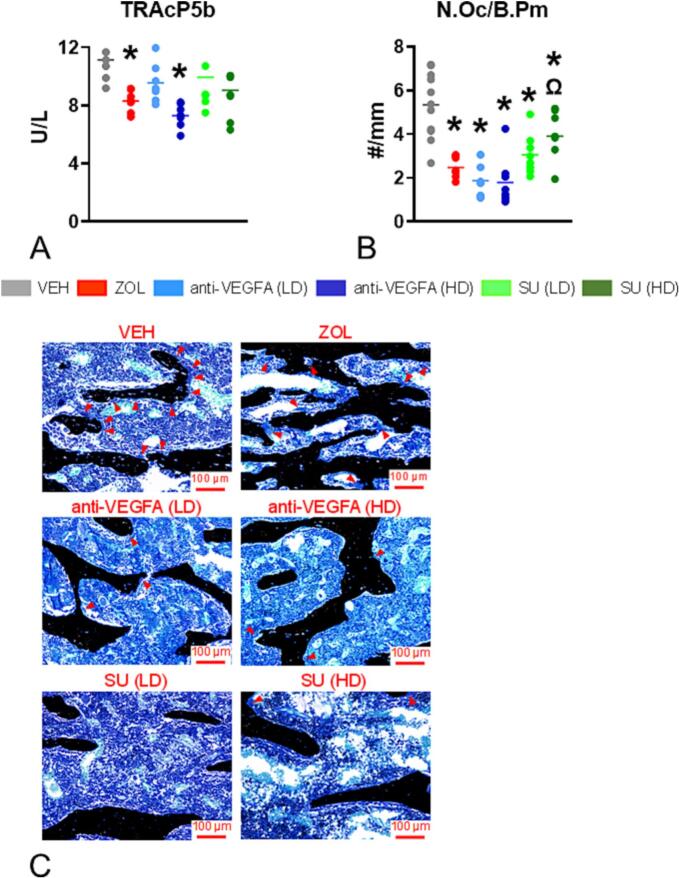
**Initial insights into an antiresorptive activity. TRAcP 5b serum levels and osteoclast number: (A)**. Serum concentration values of the bone resorption marker tartrate-resistant acid phosphatase 5b [(TRAPC5b) in units per liter (U/L); (**B**) number of osteoclasts per trabecular bone perimeter (N.Oc/B·Pm) in # per mm. Experimental groups included rats treated with vehicle (***VEH***), zoledronic acid (***ZOL)***, low or high doses of anti-VEGFA (B20–4.1.1) antibody [***anti-VEGFA*** (***LD***) and ***anti-VEGFA*** (***HD***)] IV, and low or high doses of sunitinib [***SU (LD) and SU (HD)***]. Data was analyzed by one-way ANOVA followed by Dunnett's multiple comparison tests. The data of the different groups are indicated in colored dots plots and bars (means). *Significantly different from the VEH rat group (*P* < 0.05). Ω Significantly different from the ZOL rat group (*P* < 0.05); (**C**) Representative photomicrographs depicting the osteoclasts present in trabecular bone surfaces at the distal femur metaphysis of the different rat groups. Note the greater number of osteoclasts (red arrowheads) present at trabecular bone surfaces of the VEH rat compared to the rats of the other groups.

Rats treated with ZOL (*P* = 0.0069) and anti-VEGFA (HD) (*P* = 0.0002) had significantly lower serum TRAcP 5b than VEH-treated rats (Fig. 1A). A trend for lower serum TRAcP 5b was also observed in rats treated with SU (HD) than in VEH-treated rats (*P* = 0.064). Furthermore, rats treated with ZOL (*P* < 0.0001), anti-VEGFA (LD and HD) (P < 0.0001; P < 0.0001), and SU (LD and HD) (*P* = 0.0001; *P* = 0.0239) had significantly fewer osteoclasts than VEH-treated rats at trabecular bone surfaces (Fig. 1B). In addition, rats treated with SU (HD) had significantly more osteoclasts/mm of bone surface (*P* = 0.009) than ZOL-treated rats. Fig. 1C depicts representative photomicrographs of the relative abundance of osteoclasts present in trabecular bone surfaces at the distal femur metaphysis of the different rat groups. Interestingly, as observed by Weinstein et al. (Weinstein et al., 2009) in patients receiving long-term therapy with N-BPs, giant osteoclasts with many nuclear profiles were sometimes seen at the trabecular surfaces or detached from them in ZOL-treated rats but not in the rats of the other groups (Supplemental Fig. 1C).

### 3.3 pQCT analysis

**Fig. 2:**
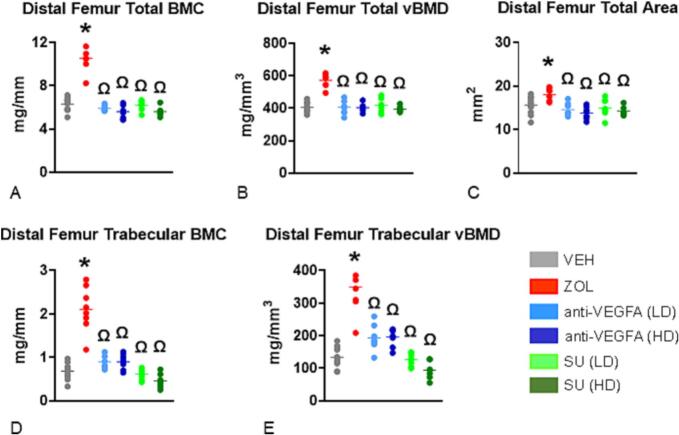
**pQCT variables at the distal femur metaphysis.** (**A**) Total bone mineral content (BMC) expressed in mg/mm; (**B**) total volumetric bone mineral density (vBMD) expressed in mg/mm^3^; (**C**) total metaphyseal area expressed in mm^2^; (**D**) trabecular BMC expressed in mg/mm; (**E**) trabecular vBMD expressed in mg/mm^3^. Data was collected at the distal femur at three levels corresponding to 25 %, 30 % and 35 % of the total bone lengths, with a starting reference line at the border of the intercondylar notch of the distal femoral epiphysis. The three levels are located at the secondary *spongiosa*, with the 25 % level closer to the growth plate and the 35 % level further away from it. The data of the pQCT variables was reported as the mean values of the three levels of measurement. Experimental groups are composed of rats treated with vehicle (***VEH***), zoledronic acid (***ZOL***), low or high doses of anti-VEGFA (B20–4.1.1) antibody [***anti-VEGFA*** (***LD***) and ***anti-VEGFA*** (***HD***)] IV, and low or high doses of sunitinib [***SU (LD) and SU (HD)***]. Data were analyzed using one-way ANOVA followed by Dunnett's multiple comparison tests. The data of the different groups are indicated in colored dots plots and bars (means). *Significantly different from the VEH rat group (*P* < 0.05). Ω Significantly different from the ZOL rat group (P < 0.05).

At the distal femoral metaphysis, rats treated with ZOL had significantly greater total BMC (*P* < 0.0001), total vBMD (*P* < 0.0001), total bone area (*P* = 0.0088), trabecular BMC (*P* < 0.0001) and trabecular vBMD (*P* < 0.0001) than VEH-treated rats (Figs. 2A-E).

**Supplemental Fig. 2:**
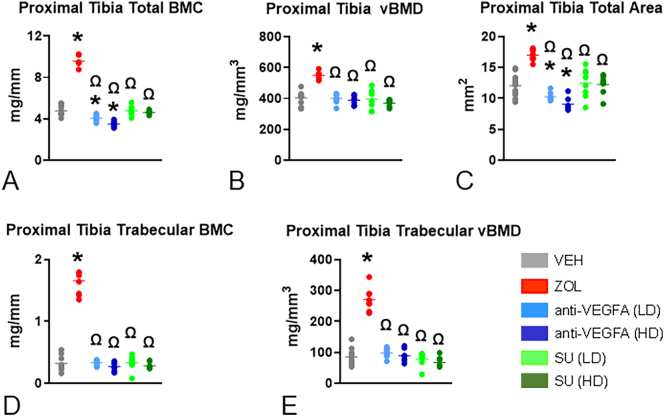
**pQCT variables at the proximal tibial metaphysis.** (**A**) Total bone mineral content (BMC) expressed in mg/mm; (**B**) total volumetric bone mineral density (vBMD) expressed in mg/mm^3^; (**C**) total metaphyseal area expressed in mm^2^ trabecular BMC expressed in mg/mm; (**C**) trabecular BMC expressed in mg/mm; (**D**) trabecular vBMD expressed in mg/mm^3^. Data were collected at the proximal tibiae at three different levels corresponding to 20 %, 25 % and 30 % of the total bone lengths with a starting reference line at the proximal tibial plateau. These levels are located at the secondary *spongiosa*, with the 20 % level closer to the growth plate and the 30 % level further away from it. Data of all pQCT variables were reported as the mean values of the three levels of measurement. Experimental groups are rats treated with vehicle (***VEH***), zoledronic acid (***ZOL)*** intravenously (IV), low or high doses of anti-VEGFA (B20–4.1.1) antibody [***anti-VEGFA*** (***LD***) and ***anti-VEGFA*** (***HD***)] IV, and low or high doses of sunitinib [***SU (LD) and SU (HD)***]. Data were analyzed using one-way ANOVA followed by Dunnett's multiple comparison test. The data of the different groups are indicated in colored dots plots and bar (means). *Significantly different from the VEH rat group (*P* < 0.05). Ω Significantly different from the ZOL rat group (P < 0.05).

Rats treated with anti-VEGFA (LD and HD) also had significantly greater trabecular vBMD than VEH-treated rats (*P* = 0.0097 and *P* = 0.0054) (Fig. 2E). Furthermore, rats treated with anti-VEGFA (LD and HD) and SU (LD and HD) had significantly lower total BMC, total vBMD, total bone area, trabecular BMC, and trabecular vBMD (all *P* < 0.0001) than ZOL-treated rats, respectively (Fig. 2A-E). At the proximal tibial metaphysis, rats treated with ZOL had greater total BMC (*P* < 0.0001), total vBMD (P < 0.0001), total bone area (P < 0.0001), trabecular BMC (P < 0.0001), and trabecular vBMD (P < 0.0001) than VEH-treated rats (Supplemental Figs. 2A-E). Rats treated with anti-VEGFA (LD and HD) had lower total BMC (*P* = 0.0027; *P* < 0.0001) and total bone area (*P* = 0.0497; *P* = 0.0003) than VEH-treated rats (Supplemental Fig. 2A and C). In addition, rats treated with anti-VEGFA (LD and HD) and SU (LD and HD) had significantly lower total BMC, total vBMD, total bone area, trabecular BMC, and trabecular vBMD (all P < 0.0001) than ZOL-treated rats (Supplemental Fig. 2A-E).

### 3.4 Histomorphometric metaphyseal structural variables

**Fig. 3:**
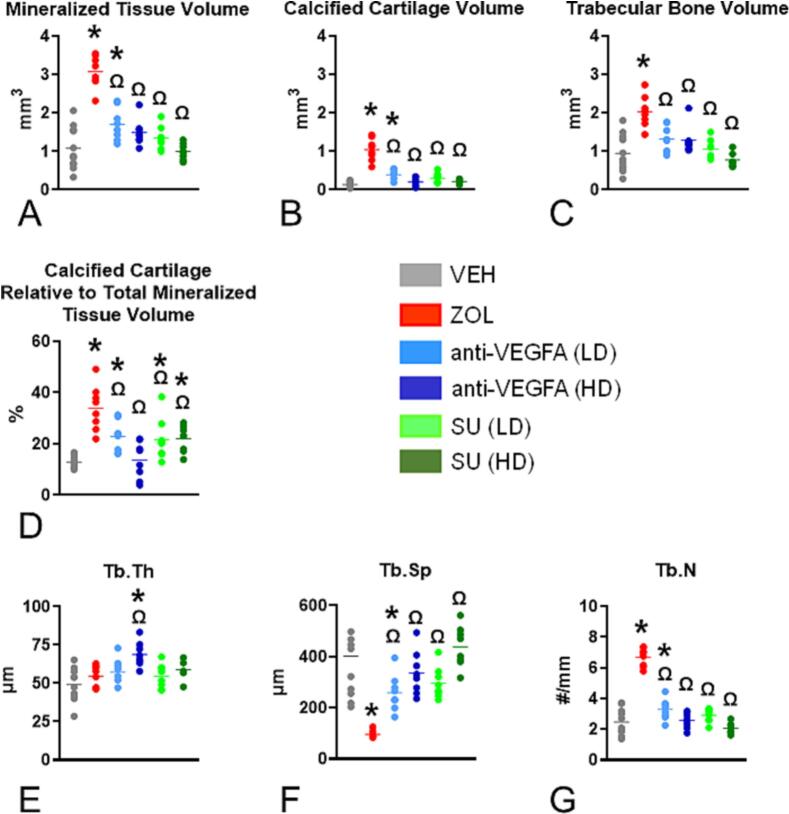
**Structural bone histomorphometric variables taken at the distal femur metaphysis**. (**A**) Mineralized tissue volume, expressed in mm^3^. This variable represents the sum of calcified cartilage volume plus trabecular bone volume. Data were collected using undecalcified sections stained with von Kossa; (**B**) Calcified cartilage volume expressed in mm^3^. Data was collected using undecalcified sections stained with toluidine blue (**C**). Trabecular bone volume expressed in mm^3^. Data were collected using undecalcified sections stained with toluidine blue; (**D**) Percentage of calcified cartilage volume relative to total mineralized tissue volume. Data were collected using undecalcified sections stained with toluidine blue; (**E)** Trabecular thickness (Tb.Th) in microns (μm), (**F**) trabecular separation (Tb.Sp) in μm; (**G**) trabecular number (Tb.N) in number (#)/μm. Experimental groups include rats treated with vehicle (***VEH***), zoledronic acid (***ZOL)***, low or high doses of anti-VEGFA (B20–4.1.1) antibody [***anti-VEGFA*** (***LD***) and ***anti-VEGFA*** (***HD***)] IV, and low or high doses of sunitinib [***SU (LD) and SU (HD)***]. Data were analyzed by one-way ANOVA followed by Dunnett's multiple comparison test. The data of the different groups are indicated in colored dots plots and bars (means). *Significantly different from the VEH rat group (P < 0.05). Ω Significantly different from the ZOL rat group (P < 0.05).

**Fig. 4:**
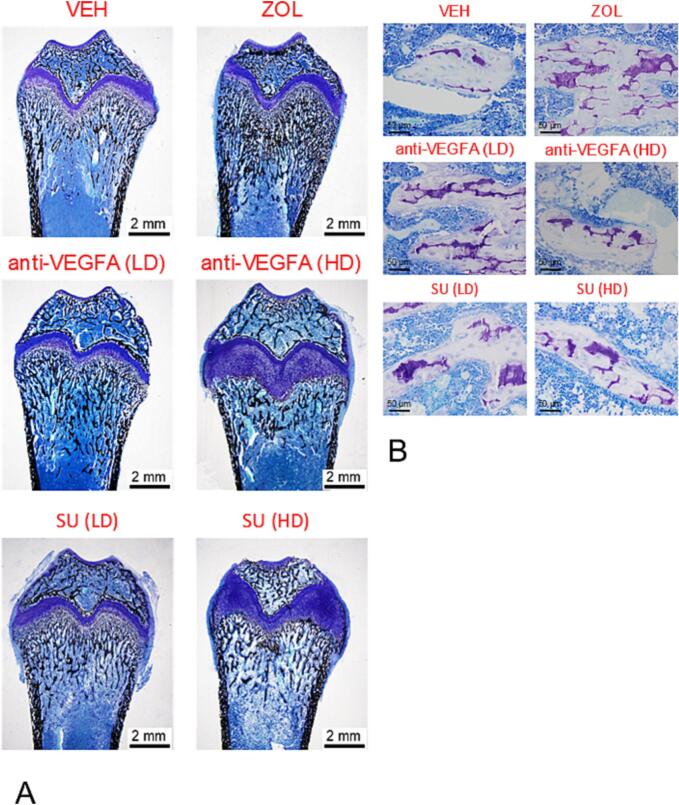
**Representative photomicrographs of metaphyseal cancellous bone tissue at the distal femur of rats of the different groups.** Distal femur metaphysis of rats treated with vehicle (VEH), zoledronic acid (ZOL), low or high doses of anti-VEGFA (B20–4.1.1) antibody [anti-VEGFA (LD) and anti-VEGFA (HD)] IV, and low or high doses of sunitinib [SU (LD) and SU (HD)]; (**A**) Sections stained with von Kossa/tetrachrome stain. Note the greater amount of cancellous bone (black-stained structures), particularly at the distal femur metaphysis in the ZOL- and anti-VEGFA (LD)-treated rats than in the VEH rat; (B) Bone trabeculae at the distal femur stained with toluidine blue stain depicting the typical metachromasia of the calcified cartilage. Note the greater amount of calcified cartilage (purple-stained structures) in contrast to the bone (lavender-stained tissue), particularly in the sections of the rats treated with ZOL, anti-VEGFA (LD), and SU (LD and HD) rats than in the VEH rat.

We observed greater mineralized tissue volume at the distal femur metaphysis in rats treated with ZOL (*P* < 0.0001) and anti-VEGFA (LD) (*P* = 0.0070) than VEH-treated rats (Fig. 3A). Fig. 4A presents photomicrographs of the distal femur metaphysis from rats in the different experimental groups, stained with von Kossa/tetrachrome, highlighting the volume of mineralized tissue observed across the different groups. We also found significantly greater calcified cartilage volume in rats treated with ZOL (*P* < 0.0001) and anti-VEGFA (LD) (*P* = 0.0023) than in VEH-treated rats (Fig. 3B). Furthermore, a trend for greater calcified cartilage volume was found in SU (LD) rats (*P* = 0.094) than in VEH-treated rats (Fig. 3B). Rats treated with ZOL (P < 0.0001) also had significantly greater trabecular bone volume than VEH-treated rats (Fig. 3C). Rats treated with anti-VEGFA (LD and HD) and SU (LD and HD) had significantly lower mineralized tissue volume (all *P* < 0.0001) and calcified cartilage volume (all P < 0.0001) than ZOL-treated rats (Fig. 3A and B). Furthermore, rats treated with anti-VEGFA (LD and HD) (*P* = 0.0002; *P* = 0.0001) and SU (LD and HD) (*P* < 0.0001; P < 0.0001) had significantly lower trabecular bone volume than ZOL-treated rats (Fig. 3C). A high trend for lower MS/BS was also observed in rats treated with ZOL (*P* = 0.0530) than VEH-treated rats. We also found a significantly greater percentage of calcified cartilage volume relative to the total mineralized tissue volume in rats treated with ZOL (*P* < 0.0001), anti-VEGFA (LD) (*P* = 0.0069) and SU (LD and HD) (*P* = 0.00211; *P* = 0.00165) than VEH-treated rats (Fig. 3D). We also found a significantly lower percentage of calcified cartilage volume relative to the total mineralized tissue volume in rats treated with anti-VEGFA (LD and HD) (*P* = 0.0137; *P* < 0.0001) and SU (LD and HD) (*P* = 0.0056; P = 0.0069) than in ZOL-treated rats (Fig. 3D). Fig. 4B depicts photomicrographs of trabeculae from the distal femur metaphysis, stained with toluidine blue, highlighting the increased amount of calcified cartilage (purple-stained structures) relative to bone tissue (lavender-stained tissue) observed in the rats treated with ZOL, anti-VEGFA (LD), and SU (both LD and HD) than in VEH-treated rats.

Furthermore, we found that rats treated with anti-VEGFA (HD) (*P* < 0.0001) had significantly greater Tb.Th than VEH-treated rats (Fig. 3E). We also observed a trend for greater Tb.The in rats treated with SU (HD) (*P* = 0.0601) than VEH-treated rats. Rats treated with anti-VEGFA (HD) (*P* = 0.0022) also had significantly greater Tb.Th than ZOL-treated rats (Fig. 3E). Moreover, rats treated with ZOL (*P* < 0.0001) and anti-VEGFA (LD) (*P* = 0.0120) had significantly lower Tb.Sp than VEH-treated rats (Fig. 3F). A trend for lower Tb.Sp was also observed in rats treated with SU (LD) (*P* = 0.0963) than VEH-treated rats. Rats treated with anti-VEGFA (LD and HD) (*P* = 0.0001; *P* < 0.0001) and SU (LD and HD) (P < 0.0001;(P < 0.0001) had greater Tb.Sp than ZOL-treated rats (Fig. 3F). In addition, rats treated with ZOL (P < 0.0001) and anti-VEGFA (LD) (*P* = 0.0249) had significantly greater Tb.N than VEH-treated rats (Fig. 3G). Rats treated with anti-VEGF (LD and HD) (P < 0.0001; P < 0.0001) and SU (LD and HD) (P < 0.0001; P < 0.0001) had significantly lower Tb.N than ZOL-treated rats (Fig. 3G).

### 3.5 Serum biomarker of bone formation

**Fig. 5:**
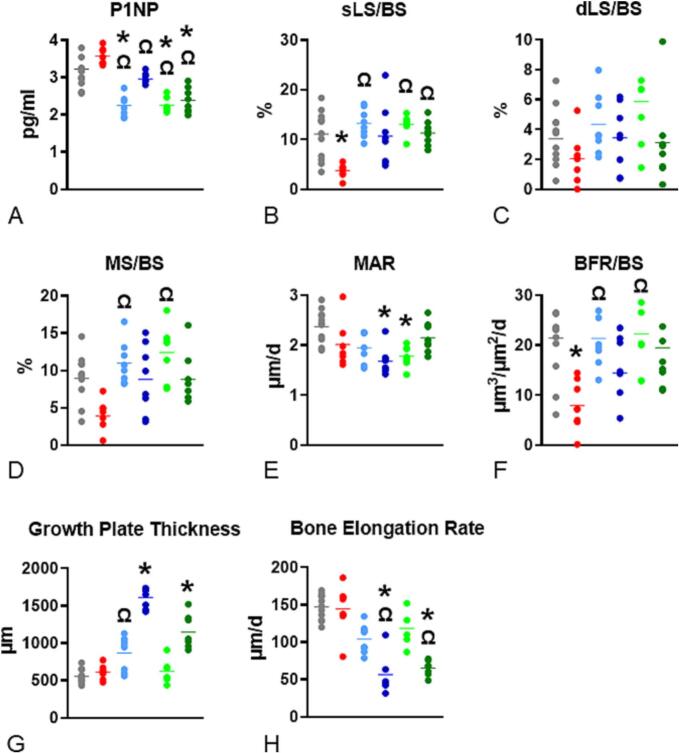
**Serum marker of bone formation, dynamic bone histomorphometry variables, growth plate thickness, & bone elongation rate at the distal femur.** (**A**) Procollagen type 1 N-terminal propeptide [(P1NP) in pg/ml; (**B**), single label per bone surface (sLS/BS) expressed in percentage; (**C**) double labels per bone perimeter (sLS/BS) expressed in percentage; (**D**) mineralizing surface per bone perimeter (MS/BS) expressed in percentage, (**E**) mineral apposition rate (MAR) expressed in μm/day; (**F**) bone formation rate per bone perimeter (BFR/BS) expressed as μm^3^/μm^2^/day (d); (**G**) The longitudinal thickness of the distal femoral epiphyseal growth plate in rat groups at the end of the study (day 10) expressed in μm; (**H**) bone elongation rate at the distal femoral metaphysis at the end of the study (day 10) expressed in μm per day. Experimental groups are rats treated with vehicle (***VEH***), zoledronic acid (***ZOL)*** intravenously (IV), low or high doses of anti-VEGFA (B20–4.1.1) antibody [***anti-VEGFA*** (***LD***) and ***anti-VEGFA*** (***HD***)] IV, and low or high doses of sunitinib [***SU (LD) and SU (HD)***]. Data was analyzed using the non-parametric Kruskal-Wallis test, followed by Dunn's multiple comparison test. The data of the different groups are indicated in colored dots plots and bars (means). *Significantly different from the VEH rat group (*P* < 0.05). Ω Significantly different from the ZOL rat group (P < 0.05).

Significantly lower serum P1NP levels were found in rats treated with anti-VEGFA (LD) (P < 0.0001) and SU (LD and HD) (P < 0.0001; P < 0.0001) than in VEH-treated rats (Fig. 5A). A trend for greater serum P1NP was found in rats treated with ZOL than in VEH-treated rats (*P* = 0.078). Rats treated with anti-VEGFA (LD and HD) (P < 0.0001; P < 0.0001) and SU (LD and HD) (P < 0.0001; P < 0.0001) had significantly lower serum P1NP levels than ZOL-treated rats (Fig. 5A).

### 3.6 Dynamic bone histomorphometry

Rats treated with ZOL had lower sLS/BS than VEH-treated rats (*P* = 0.0088) (Fig. 5B). Rats treated with anti-VEGFA (LD) (*P* = 0.0012) and SU (LD and HD) (*P* = 0.0027; *P* = 0.049) had greater sLS/BS than ZOL-treated rats. No significant differences in dLS/BS were found between any of the treatment groups and VEH-treated rats (Fig. 5C). Rats treated with anti-VEGFA (LD) (*P* = 0.0039) and SU (LD) (*P* = 0.0010) had significantly higher MS/BS than ZOL-treated rats. (Fig. 5D). Rats treated with anti-VEGFA (HD) (*P* = 0.0008) and SU (LD) (*P* = 0.0198) had significantly lower MAR than VEH-treated rats (Fig. 5E). Furthermore, BFR/BS was significantly lower in rats treated with ZOL (*P* = 0.0059) than in VEH-treated rats (Fig. 5F). In contrast, rats treated with anti-VEGFA (LD) (*P* = 0.0222) and SU (LD) (*P* = 0.0278) had significantly greater BFR/BS than ZOL-treated rats (Fig. 4F).

### 3.7 Growth plate thickness and bone elongation rate

Rats treated with anti-VEGFA (HD) (*P* ≤0.0001) and SU (HD) (*P* = 0.0018) had significantly thicker growth plates than VEH-treated rats (Figs. 5G & 4A). Rats treated with anti-VEGFA (HD) (*P* = 0.011) had significantly thicker growth plates than ZOL-treated rats (Fig. 5G). A trend for greater bone elongation rate was also observed in rats treated with SU (HD) (*P* = 0.083) compared to rats treated with ZOL (Fig. 5G). Rats treated with anti-VEGFA (HD) (*P* = 0.0002) and SU (HD) (*P* = 0.0004) had a significantly lower rate of bone elongation than VEH-treated rats (Fig. 5H). Rats treated with anti-VEGFA (HD) (*P* = 0.0014) and SU (HD) (*P* = 0.0026) had significantly lower bone elongation rates than ZOL-treated rats (Fig. 5H).

## 4 Discussion

While AgIs are suspected systemic risk factors for MRONJ, their precise role is not as well-defined as that of pARs. This uncertainty arises mainly because systemic AgIs are never used alone in clinical settings; they are always combined with other anti-neoplastic drugs (Nicolatou-Galitis et al., 2019; Pimolbutr et al., 2018; Katsenos et al., 2012; Disel et al., 2012; Estilo et al., 2008; Greuter et al., 2008; Guarneri et al., 2010; Serra et al., 2009) or pARs (Aragon-Ching et al., 2009; Christodoulou et al., 2009; Lescaille et al., 2014; Ngamphaiboon et al., 2011). The leading hypothesis for the involvement of systemic risk factors in MRONJ pathophysiology focuses on the inhibition of bone resorption, which primarily supports the role of pARs but does not explain how AgIs might contribute to MRONJ (Ruggiero et al., 2022; Khosla et al., 2007; Bilezikian, 2006; Aragon-Ching et al., 2009; Nicolatou-Galitis et al., 2019; Pimolbutr et al., 2018; Koch et al., 2011; Katsenos et al., 2012; Disel et al., 2012; Estilo et al., 2008; Greuter et al., 2008; Guarneri et al., 2010; Serra et al., 2009; Aguirre et al., 2021). The current hypothesis regarding AgIs relates to their primary action of inhibiting angiogenesis. Notably, various AgIs, including anti-VEGFA, SU, and DA, have been shown to inhibit osteoclast formation and activity both *in vitro* and *in vivo* (Maita et al., 2012; Murray et al., 2003; Davis et al., 2011; Miyazaki et al., 2004; Park et al., 2019; Lowe et al., 1993; Niida et al., 2005; Niida et al., 1999; Patyna et al., 2008; Zhang et al., 2014). Our data on serum TRACP 5b and osteoclast numbers in rats treated with anti-VEGFA and SU aligns with these findings, raising the question of whether AgIs themselves have sufficient effects on endpoints reflecting *in vivo* bone resorption to be considered antiresorptive agents. Currently, no studies compare the *in vivo* anti-resorptive activity of clinical doses of AgIs to pARs. A significant portion of the bone resorption data in this study was generated using the Schenk assay, a preclinical model regarded as a “*gold standard*” for evaluating the antiresorptive activity of drugs (Schenk et al., 1986; Schenk et al., 1973). This assay has been employed in drug discovery for over 50 years (Schenk et al., 1986; Schenk et al., 1973). We initially utilized undecalcified distal femur sections stained with von Kossa, which reveals all calcified tissues and calcium deposits. The von Kossa method's silver cations react with calcium in both bone and calcified cartilage, producing the characteristic black stain of mineralized tissues. The growing long bone metaphysis typically experiences net hard tissue loss, which results from the net activity of resorption of calcified cartilage and bone by osteoclasts and bone formed by osteoblasts. Woven bone is formed in the metaphysis at the surfaces of calcified cartilage in the primary *spongiosa* and at the bone surfaces in the secondary spongiosa. When bone resorption is inhibited, such as with pARs, the total mineralized tissue in the metaphysis increases, serving as a reliable index of bone resorption activity (Miller and Jee, 1975; Schenk et al., 1986; Schenk et al., 1973). As expected, rats treated with an oncologic dose of ZOL, a representative of the N-BP class, exhibited ~3 times more von Kossa-positive mineralized tissue volume than VEH-treated rats. Rats treated with anti-VEGFA (both LD and HD) showed mild retention of mineralized tissue in the trabeculae, about 1.5 times that of VEH-treated rats, but significantly less than in ZOL-treated rats. In contrast, rats treated with SU (both LD and HD) demonstrated no differences in mineralized tissue volume than VEH rats, and they had significantly less than ZOL-treated rats. Thus, while anti-VEGFA shows substantial anti-resorptive activity, it is notably lower than that of an oncology dose of ZOL. A portion of the metaphyseal mineralized tissue comprises calcified cartilage, which is a remnant of the mesenchymal tissues left behind by the advancing growth plate during bone elongation. Unlike bone, calcified cartilage is only resorbed and not formed in the metaphysis of a growing long bone, making its quantification reliant on histomorphometric techniques and specific stains that differentiate between bone and calcified cartilage. When assessing the antiresorptive activity of an agent, the amount of calcified cartilage in the metaphysis of a growing rat serves as a crucial indicator of resorptive activity. The quantity of calcified cartilage is maximal in the primary spongiosa and decreases toward the diaphysis, as osteoclastic resorptive activity has typically acted on the mineralized tissue in the metaphysis. Bone modeling is the biological process that removes calcified cartilage and woven bone, transforming it into lamellar bone in the mature metaphysis. This coordinated activity involves the resorption of mineralized tissue by osteoclasts and the concurrent formation of new bone by osteoblasts. All calcified cartilage in the metaphysis arises from the orderly degradation of growth cartilage, which preserves longitudinal calcified cartilage septae. To analyze the various morphological components of the metaphyseal mineralized tissues, consecutive undecalcified frontal sections of the distal femur were stained with toluidine blue. The volumes of calcified cartilage and trabecular bone were recorded separately. We found that rats treated with ZOL had a calcified cartilage volume 7.7 times greater than that of VEH-treated rats.

**Supplemental Fig. 3:**
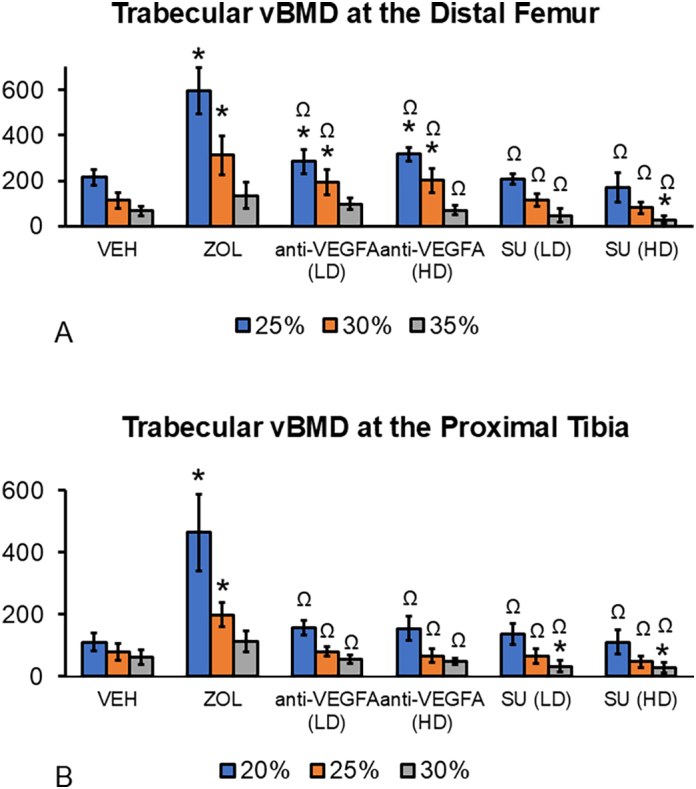
**Trabecular vBMD at the distal femur and proximal tibia were determined at three different levels of each metaphysis.** (**A**) Total volumetric bone mineral density (vBMD) at the distal femur metaphysis expressed in mg/mm^3^. Data were collected at the distal femurs at three different levels corresponding to 25 %, 30 % and 35 % of the total bone lengths, with a starting reference line at the border of the intercondylar notch of the distal epiphysis of the femurs. The three levels are located at the secondary *spongiosa*, with the 25 % level closer to the growth plate and the 35 % level further away from it; (**B**) Total vBMD at the proximal tibial metaphysis expressed in mg/mm^3^. Data were collected at the proximal tibias at three different levels corresponding to 20 %, 25 % and 30 % of the total bone lengths with a starting reference line at the proximal tibial plateau. These levels are located at the secondary *spongiosa*, with the 20 % level closer to the growth plate and the 30 % level further away from it. Experimental groups are composed of rats treated with vehicle (***VEH***), zoledronic acid (***ZOL)*** intravenously (IV), low or high doses of anti-VEGFA (B20–4.1.1) antibody [***anti-VEGFA*** (***LD***) and ***anti-VEGFA*** (***HD***)] IV, and low or high doses of sunitinib [***SU (LD) and SU (HD)***]. Data were analyzed using one-way ANOVA followed by Dunnett's multiple comparison test. The data of the different groups are indicated by colored bars (means) ± standard deviation (SD). *Significantly different from the VEH group (P < 0.05). Ω Significantly different from the ZOL group (P < 0.05).

Additionally, rats treated with anti-VEGFA (LD) but not anti-VEGFA (HD) exhibited a calcified cartilage volume ~ 3 times greater than that of VEH-treated rats. In contrast, SU-treated rats showed no differences in these measures. The remaining metaphyseal mineralized tissue is trabecular bone, calculated by subtracting the volume of calcified cartilage from the total mineralized tissue volume. Trabecular bone first appears in the metaphysis as woven bone near the growth cartilage–metaphyseal junction (GCMJ). It gradually transitions to lamellar bone as woven bone is resorbed, and new lamellar bone is formed at the metaphyseal surfaces. While differentiating between woven and mature lamellar tissue could have provided additional insight, this study did not address this distinction. We found that ZOL rats had greater trabecular bone volume than VEH rats, while no differences were found among the other groups. Furthermore, ZOL rats exhibited a higher calcified cartilage-to-bone ratio than VEH-treated rats. Rats treated with anti-VEGFA (LD) and SU (LD and HD) also showed greater calcified cartilage-to-bone ratios, albeit to a lesser extent than ZOL rats. These findings suggest two possible interpretations regarding anti-VEGFA and SU effects; they may: 1) inhibit osteoclast activity, as calcified cartilage is exclusively resorbed in the metaphysis, or 2) decrease trabecular bone formation, potentially due to inhibited bone elongation and osteoblast activity. We conclude that anti-VEGFA and SU, at doses comparable to those used in cancer patients, may have anti-resorptive activity, though significantly less than that of an oncology dose of ZOL. The pQCT analysis confirmed the significant effects of an oncologic dose of ZOL in retaining mineralized tissue at the distal femoral metaphysis, indicated by greater total BMC, vBMD, bone area, trabecular BMC, and trabecular vBMD at both the distal femur (Fig. 2) and proximal tibia (Supplemental Fig. 2). In contrast, anti-VEGFA (LD and HD) resulted in only a mild but significant increase in trabecular vBMD in the distal femoral metaphysis (Fig. 2), with no change in the proximal tibia (Supplemental Fig. 2). Rats treated with SU (LD and HD) exhibited lower trabecular vBMD in the distal femur (35 % decrease) and proximal tibia (30 % decrease) (Supplemental Fig. 3). Taken together, these data moderately align with the serum TRAcP 5b levels data, which shows lower values in rats treated with ZOL and anti-VEGFA (HD) and a tendency to have lower values in the SU (HD) rats. Further, all rat groups had fewer osteoclasts (N.Oc/B·Pm) than the VEH rats. These data are consistent with the findings on the bone resorption variables for rats treated with ZOL and, to some extent, for the anti-VEGFA rats. The low osteoclast numbers in SU rats may account for lower TRAcP5b levels and higher calcified cartilage-to-bone ratios observed in SU rats. These findings align with Maita et al. (Maita et al., 2012), who reported fewer TRAP+ cells in tumor-bearing mice treated with SU. Conversely, the lower osteoclast number in SU rats appeared to be inconsistent with other bone resorption indices, giving an initial false impression of lacking coherence. One possible explanation is that SU may reduce the bone elongation rate, as indicated by increased growth plate thickness (Poterucha et al., 2012). This explanation may also apply to the anti-VEGFA groups. Our study also observed lower serum P1NP in rats treated with SU and anti-VEGFA (LD), consistent with the reduced MAR, a reliable measure of osteoblast function (Recker et al., 2011). Thus, a lower MAR suggests reduced osteoblast activity (Parfitt et al., 1987; Blue et al., 2015; Gregory et al., 2013; Yirmiya et al., 2006). Although anti-VEGFA and SU treatments led to a lower osteoclast number, the increase in metaphyseal mineralized volume was minimal due to concurrent decreases in osteoblast activity and bone elongation rates. As expected for an N-BP (Jensen et al., 2021; Eriksen et al., 2002; Bravenboer et al., 1999; Chavassieux et al., 1997; Ste-Marie et al., 2004), ZOL-treated rats exhibited lower BFR/BS, primarily due to a decrease in MS/BS (high trend), a surrogate for osteoblast number (Dempster et al., 2013). Consistent with previous studies (Boyce et al., 2014; Baroncelli and Bertelloni, 2014; Brumsen et al., 1997), ZOL did not affect the rate of bone elongation. Thus, the high trend in reduced MS/BS can be attributed to the significant suppression of metaphyseal bone remodeling resulting from ZOL's inhibition of bone resorption. Our study also found that ZOL does not affect serum P1NP levels in growing rats, which is in contrast to many studies in osteoporosis patients that report decreased PINP concentrations following N-BP exposure. However, our results align with some short-term studies on N-BPs that also observed no changes in serum P1NP (Nakamura et al., 2021; Liu et al., 2021). The lack of variation in P1NP levels may be due to the brief duration of our study, which might not have allowed sufficient time to affect this marker. Typically, serum bone resorption markers decline first, followed by a more gradual decrease in bone formation markers (Szulc, 2020). Furthermore, ZOL's lack of effect on serum P1NP might be explained by the fact that the metaphysis of growing long bones, particularly the primary spongiosa, undergoes bone modeling rather than remodeling. In bone modeling, resorption and formation can occur simultaneously but at different sites, unlike in adult remodeling, where resorption precedes formation. Consequently, lower serum P1NP levels in N-BP-treated adults may be due to a reduced activation frequency of new bone remodeling units rather than a direct effect of ZOL on osteoblasts. In contrast, the lower serum P1NP levels in rats treated with anti-VEGFA (LD) and SU (LD and HD) correlated with a reduced rate of bone elongation. The decreased MAR in the distal femoral metaphysis suggests that P1NP levels in these groups reflect impacts on bone elongation and osteoblast activity rather than the number of active osteoblasts.

We observed physeal dysplasia in the distal femur of rats treated with anti-VEGFA (HD) and SU (HD), characterized by uneven thickening of the growth plate. Anti-VEGFA treatment resulted in a dose-related increase in the hypertrophic chondrocyte layer. These findings correspond with studies in cynomolgus monkeys receiving BVZ (Ryan et al., 1999). In addition, rats treated with SU (HD) exhibited growth plate thickening, consistent with prior studies in rats and monkeys (Patyna et al., 2008). Finally, rats treated with anti-VEGFA (HD) experienced lower body weight gain than VEH rats, paralleling similar findings in cancer patients treated with BVZ (Poterucha et al., 2012).

We acknowledge several limitations in this study. *First*, our research focused on only two AgIs, which may limit the applicability of the findings to other agents. *Second*, we examined only two doses of anti-VEGFA and SU, restricting our ability to establish a dose-response relationship. *Third*, while the study demonstrates mild anti-resorptive effects, it does not investigate the underlying molecular mechanisms, necessitating further research. *Fourth*, we utilized only an oncologic dose of ZOL (80 μg/kg) as a positive control, which may not fully represent the effects of an osteoporosis dose (8 μg/kg). Including the lower dose could have provided a more comprehensive view of the relative antiresorptive capacity of the tested AgIs. *Fifth*, the 10-day duration of the Schenk assay offers only a short-term estimate of anti-resorptive properties, limiting our ability to assess long-term adaptations. *Sixth*, the final fluorochrome label was injected only one day before euthanasia. This short time interval (typically 2–3 days) may have allowed for partial washout of the label, resulting in an underestimation of the double-labeled surface and affecting the accuracy of the bone elongation rate measurements. *Finally*, while we primarily investigated anti-resorptive effects, additional long-term *in vivo* studies are needed to comprehend the complete impact of these drugs on bone cell activities, including modeling, remodeling, and bone elongation. In conclusion, our study shows that these AgIs, particularly anti-VEGFA, exhibit significant but milder anti-resorptive activity than the oncologic dose of ZOL. This is supported by reduced osteoclast numbers on trabecular bone surfaces and increased calcified cartilage volume at the distal femur metaphysis. In addition, anti-VEGFA lowered serum TRACP-5b levels and increased mineralized tissue volume, albeit less than ZOL. Both AgIs decreased MAR, serum P1NP levels, and bone elongation rates while increasing growth cartilage thickness and inducing physeal dysplasia. These inhibitory effects on bone formation may further contribute to the pathophysiology of MRONJ, especially during the healing phase.

The following are the supplementary data related to this article.

## Abbreviations

MRONJ: Medication-related osteonecrosis of the jaw
pARs: Powerful antiresorptives
N-BPs: Nitrogen-containing bisphosphonates
ZOL: Zoledronic acid
AgIs: Angiogenesis inhibitors
TKIs: Multitargeted receptor tyrosine kinase inhibitors
BVZ: Bevacizumab
anti-VEGFA: Anti-Vascular Endothelial Growth Factor A Antibody
SU: Sunitinib
VEGFRs: Vascular endothelial growth factor receptors
PDGFRs: Platelet-derived growth factor receptors
CSF1R: Colony stimulating factor 1 receptor
c-SRC: Proto-oncogene tyrosine-protein kinase Src
VEH: Vehicle
LD: Low dose
HD: High dose
TRAPC5b P1NP: Tartrate-resistant acid phosphatase 5b
P1NP: Procollagen type 1 N-terminal propeptide
pQCT: Peripheral Quantitative Computed Tomography
BMC: bone mineral content
vBMD: volumetric bone mineral density
ROI: Region of interest
GCMJ: Growth cartilage-metaphyseal junction
BV/TV: Bone volume fraction
Tb. Th: Trabecular thickness
Tb. S: Trabecular separation
Tb.N: Trabecular number
Oc.N/BS: Osteoclast number per bone surface
sL/BS: Single-labeled surfaces
dL/BS: Double-labeled surfaces
MS/BS: Mineralizing surface
MAR: Mineral apposition rate
BFR/BS: Bone formation rate

## CRediT authorship contribution statement

**J.I. Aguirre:** Conceptualization, Data curation, Formal analysis, Funding acquisition, Investigation, Methodology, Project administration, Supervision, Validation, Writing – original draft, Writing – review & editing. **S.M. Croft:** Data curation, Formal analysis, Methodology, Writing – review & editing. **E.J. Castillo:** Data curation, Formal analysis, Software, Writing – review & editing. **C.J. Cruz-Camacho:** Data curation, Investigation, Methodology, Writing – review & editing. **D.B. Kimmel:** Conceptualization, Data curation, Investigation, Supervision, Writing – review & editing.

## Publisher's note

All claims expressed in this article are solely those of the authors and do not necessarily represent those of their affiliated organizations or those of the publisher, the editors and the reviewers. Any product that may be evaluated in this article or claim that its manufacturer may make is not guaranteed or endorsed by the publisher.

## Ethics statement

The experimental animal protocol was reviewed and approved by the UF Institutional Animal Care and Use Committee (IACUC).

## Funding

This research was supported by the 10.13039/100000072National Institute of Dental and Craniofacial Research (NIDCR); R01DE023783-01A and a Seed Grant from the 10.13039/100012651College of Veterinary Medicine, University of Florida.

## Declaration of competing interest

The authors have no conflicts of interest.

## Data Availability

The raw data supporting the conclusions of this article will be made available by the authors upon request.
